# The Effect of Root Exudate 7,4′-Dihydroxyflavone and Naringenin on Soil Bacterial Community Structure

**DOI:** 10.1371/journal.pone.0146555

**Published:** 2016-01-11

**Authors:** Márton Szoboszlay, Alison White-Monsant, Luke A. Moe

**Affiliations:** 1 Department of Plant and Soil Sciences, University of Kentucky, Lexington, Kentucky, United States of America; 2 Department of Animal, Plant and Soil Science, Centre for AgriBioscience, La Trobe University, Melbourne, Australia; Graz University of Technology (TU Graz), AUSTRIA

## Abstract

Our goal was to investigate how root exudate flavonoids influence the soil bacterial community structure and to identify members of the community that change their relative abundance in response to flavonoid exudation. Using a model system that approximates flavonoid exudation of *Medicago sativa* roots, we treated a soil with 7,4′-dihydroxyflavone and naringenin in two separate experiments using three different rates: medium (equivalent to the exudation rate of 7,4′-dihydroxyflavone from *M*. *sativa* seedlings), high (10× the medium rate), and low (0.1× the medium rate). Controls received no flavonoid. Soil samples were subjected to ATP assays and 16S rRNA gene amplicon sequencing. The flavonoid treatments caused no significant change in the soil ATP content. With the high 7,4′-dihydroxyflavone treatment rate, operational taxonomic units (OTUs) classified as *Acidobacteria* subdivision 4 increased in relative abundance compared with the control samples, whereas OTUs classified as *Gaiellales*, *Nocardioidaceae*, and *Thermomonosporaceae* were more prevalent in the control. The naringenin treatments did not cause significant changes in the soil bacterial community structure. Our results suggest that the root exudate flavonoid 7,4′-dihydroxyflavone can interact with a diverse range of soil bacteria and may have other functions in the rhizosphere in addition to *nod* gene induction in legume—rhizobia symbiosis.

## Introduction

Flavonoids are plant secondary metabolites synthesized via the phenylpropanoid pathway. They are present in all tissues of higher plants and some are exuded from the roots into the rhizosphere [1]. Their most studied functions in the soil are those associated with legume—rhizobia symbiosis, whereby they activate or repress bacterial *nod* gene expression [2] and trigger chemotaxis in nitrogen-fixing rhizobia [3]. Aside from their association with rhizobia, flavonoids are present in root exudates of non-legume plants [1], and a growing body of data suggests that they influence the growth and activity of various soil bacteria. For example, some flavonoids, particularly isoflavonoids, are considered phytoalexins or phytoanticipins due to their antimicrobial effect [4]. Furthermore, the experiments of Hartwig et al. [5] imply that root exudate flavonoids may control the proliferation of some rhizosphere bacteria. They found that the doubling time of *Ensifer meliloti* and *Pseudomonas putida* decreased when exposed to luteolin or quercetin in micromolar concentrations in laboratory cultures. Flavonoids can also be utilized as a source of carbon and have other direct and indirect effects on soil nutrient cycles [6]. Root extracts with a high flavonoid content from *Lupinus albus* have been shown to decrease soil respiration without a significant change in microbial biomass based on soil ATP content, and to decrease phosphatase and increase urease activity [7]. Flavonoids may also affect bacterial activity in the rhizosphere by influencing quorum sensing. Vandeputte et al. [8,9] found that catechin, apigenin, eridictyol, kaempferol, luteolin, myricetin, naringenin, naringin, quercetin, taxifolin, and chalcone had some effect on the production of quorum-sensing-dependent factors in *Pseudomonas aeruginosa*. Further, Pérez-Montaño et al. [10] showed that acyl-homoserine-lactone production by *Ensifer fredii*, *Rhizobium etli*, and *R*. *sullae* strains was enhanced in the presence of flavonoids that are known to induce expression of their cognate *nod* genes.

These findings indicate that root exudate flavonoids influence a diverse range of soil bacteria. To improve our understanding of how root exudate flavonoids influence the soil microbial community structure, we designed a model system that approximates flavonoid exudation of *Medicago sativa* roots. Our goal was to identify members of the soil bacterial community that change their relative abundance in response to flavonoid exudation. To achieve this, we explored the impact of simulated exudation of 7,4′-dihydroxyflavone, the most abundant *nod* gene inducing flavonoid among the root exudates of *M*. *sativa* seedlings [11], and naringenin, which is also a *nod* gene inducing flavonoid present in the root exudates of various legumes [1].

## Materials and Methods

### Soil properties

The soil chosen for the rhizosphere model system studies was from a pasture at the University of Kentucky Spindletop Farm (38°06'51.7"N, 84°29'41.7"W) that had not been fertilized or planted for the last 5 years; but prior to this, *M*. *sativa* had been grown for the study by Probst and Smith [12]. The Maury silt loam (fine, mixed, semiactive, mesic Typic Paleudalf) was collected from the surface 10–15 cm, sieved (4 mm), dried at room temperature with regular mixing, and then stored at room temperature in a closed plastic container for over 3 months. This storage period was to decrease the effect of root exudate flavonoids in the soil from the vegetation at the collection site. Samples were sent to the University of Kentucky Regulatory Services Soil Testing Laboratory to determine basic soil properties (http://soils.rs.uky.edu/tests/methods.php).

The soil texture was 18.5% sand, 64.1% silt, and 17.4% clay. The pH was 5.95 (1:1 solution:soil using 1 M KCl) and the buffered pH was 7.03 (with Sikora buffer [13]). The soil contained 4.49% organic matter and 0.25% total nitrogen. The cation exchange capacity was 25.95 meq/100 g, the base saturation 87.77% and the exchangeable K, Ca, Mg, and Na were 0.36, 17.85, 4.55, and 0.02 meq/100 g. Mehlich III extractable P, K, Ca, Mg, and Zn were 243.5, 110.0, 2925.5, 486.5, and 1.2 mg/kg.

Before using the soil it was mixed with 30% m/m sand (previously bleached, washed, and oven dried) to facilitate drainage.

### The rhizosphere model system

The soil—sand mixture was moistened with distilled water (60.0 g water to 400.0 g soil—sand mixture) and thoroughly mixed in plastic bags before filling 60.0 g into 50-ml polypropylene conical centrifuge tubes (Fisher Scientific, USA). The conical bottom portions of the tubes were previously cut off and replaced with plastic mesh to allow drainage. Each tube was wrapped in aluminum foil to protect the soil from light. We used rhizon soil moisture samplers (Rhizon MOM, Rhizosphere Research Products, Netherlands) to model the root exudation process. The rhizons had 5-cm long porous parts, 2.5 mm diameter, pore size 0.12–0.18 μm, a glass fiber strengthener, and polyethylene/polyvinylchloride tubing. To the soil in each tube, 6.00 ml distilled water was added before inserting three rhizons vertically and equidistant to each other and the wall of the tube. The rhizons were previously bleached and then washed in sterile distilled water.

Initial experiments studying the effect of root exudates on the soil microbial community delivered exudates to the soil via artificial roots such as membrane filters [14,15] or cylindrical tubes or wicks prepared from such filters [16,17]. In later experiments, rhizon soil moisture samplers were employed as root exudation models [18–20] by using them in the reverse direction: to pump a solution into the soil instead of sampling the soil solution. An alternative approach developed by Ziegler et al. [21] used glass slides coated with agarose containing the exudate compounds that were inserted into the soil. Our goal was to study the microbial community in a volume of soil homogenously exposed to the exudates; therefore, we adopted the use of rhizon soil moisture samplers in our rhizosphere model systems.

### Treatment solutions and applications

The rhizosphere model systems received 7,4′-dihydroxyflavone or naringenin at high (24.00 nmol/day), medium (2.40 nmol/day), low (0.24 nmol/day), or no flavonoid (control) rates. The medium treatment rate was determined from the calculations of Cesco et al. [1] which were based on the results of Maxwell and Phillips [11] to match the exudation rate of 7,4′-dihydroxyflavone from *M*. *sativa* seedlings assuming that 0.4 g (fresh weight) root biomass occupies the soil in a rhizosphere model system. We determined the root biomass value experimentally by growing *M*. *truncatula* using the same soil and tube setup as the present experiment (unpublished). The treatment solutions were applied in 1.2 ml aqueous volume slowly pumped through the rhizons (400 μl per rhizon) into the soil using 1-ml syringes, once every 24 hours. After each application, approximately 0.2 ml of air was injected into the rhizon to ensure that the entire volume of the treatment solution had reached the soil and that no liquid remained in the tubing.

Stock solutions of 7,4′-dihydroxyflavone (Indofine, USA) and naringenin (MP Biomedicals, USA) were prepared in dimethyl sulfoxide and stored at −20°C until the start of the treatments. The final treatment solutions each contained 0.0625% v/v dimethyl sulfoxide. The treatment solutions for each flavonoid treatment rate and the control were supplemented with the major carbohydrates, amino acids, and other organic acids present in the root exudates of *M*. *sativa* [22–24] assuming 0.4 g (fresh weight) root biomass in the rhizosphere model system: 1396 μM glucose, 223 μM arabinose, 115 μM maltose, 108 μM mannose, 690 μM serine, 250 μM glycine, 26 μM malate, 24 μM citrate, and 4 μM succinate. Stock solutions were prepared in water, filter sterilized (0.22 μm), and stored at 4°C. The control treatments received the carbohydrates, amino acids, and other organic acids, and dimethyl sulfoxide, but no flavonoid. The treatment solutions were adjusted to pH 7 with KOH.

Six rhizosphere model systems were set up for each of the high, medium, low, and control treatment rates, and then placed on plastic trays and incubated in a dark cabinet at room temperature (21–23°C). A 400-μl aliquot of distilled water was pumped through each rhizon once a day for 5 days before the treatments started. In the first days of incubation a few seedlings germinated in the soil. This affected 3 rhizosphere model systems receiving the high 7,4′-dihydroxyflavone rate, 1 receiving the medium rate, 3 receiving the low rate and 2 receiving the control treatment. From the naringenin treatments 2 rhizosphere model systems receiving the high rate, 1 receiving the medium rate, 1 receiving the low rate, and 2 receiving the control treatment were affected. Seedlings were removed as soon as they emerged from the soil. The last seedling appeared 6 days before sampling. The treatments were applied for 10 days. Every 2 days the rhizosphere model systems were weighed before administering the treatments and then sufficient distilled water was applied to the soil to maintain a constant water content, and they were randomly rearranged on the trays.

The experiment was first conducted with 7,4′-dihydroxyflavone and then repeated with naringenin using the same procedures.

### Sampling

Soil samples were harvested approximately 3 hours after the last treatment application. The rhizons were removed and the soil column was pushed out of the tube. The top 1 cm of soil was discarded and the next 4 cm was collected in a sterile plastic bag using a sterile spatula, mixed, and then frozen in liquid nitrogen. Samples were stored at −80°C prior to analysing all samples for microbial biomass and microbial community structure.

Some rhizosphere model systems contained germinating seeds that did not reach the surface, and thus were only detected during sampling. These systems were removed from the study leaving the number of replicates to 5 high, 4 medium, 5 low, and 4 control in the 7,4′-dihydroxyflavone experiment, and 5 high, 5 medium, 4 low, and 5 control in the naringenin experiment.

### ATP assays

To assess total microbial biomass ATP was extracted from 1.0 g of the soil samples with dimethyl sulfoxide and trisodium phosphate as described by Bai et al. [25], but with 1:10 instead of 1:100 dilution in glycine-EDTA buffer. Diluted extracts were treated with benzalkonium chloride following the protocol of Martens [26] by mixing a 100-μl aliquot with 100 μl 0.05% m/m benzalkonium chloride in Tris-Mg^2+^ buffer (50 mM Tris, 10 mM MgSO_4_, pH 7.8) in a 12 mm × 50 mm autoclaved glass tube. The tube was sonicated for 5 s and 190 μl Tris-Mg^2+^ buffer was added, followed by 10 μl StayBrite Highly Stable Luciferase/Luciferin Reagent (BioVision, USA). The sample was quickly mixed with a pipet and the luminescence was measured five consecutive times using a Turner Design 20/20 luminometer with 10-s integration periods and 54.8% sensitivity. Out of the five measurements, generally the first two were higher than the last three, which showed less variation. Therefore, the average of the third, fourth, and fifth readings was used in the calculation. Blanks were used to measure the background luminescence using glycine-EDTA buffer instead of a soil extract. A standard curve using log_10_ (ATP concentration in nM) vs. log_10_ (luminescence) was obtained by measuring ATP standards comprising blank samples supplemented with 0.1, 0.5, 1, 5, and 10 nM ATP. The ATP stock solution was prepared from ATP-Na_2_ salt (SERVA Electrophoresis GmbH, Germany) in Tris-Mg^2+^ buffer and then filter sterilized (0.22 μm) and stored at −80°C. To estimate the extraction efficiency, soil samples from the 7,4′-dihydroxyflavone (n = 3) and the naringenin (n = 3) experiments were randomly chosen, and ATP extracts were prepared from them by adding 20 μl 0.1 mM ATP solution when adding the dimethyl sulfoxide.

Samples from the 7,4′-dihydroxyflavone and naringenin experiments were processed separately. Three blanks and a set of ATP standards were measured for both experiments. The average of the blanks was subtracted from the readings and the ATP content per gram of soil (dry weight) was calculated according to the standard curves in Microsoft Excel. The results were analyzed in JMP 10.0.0 (SAS Institute, USA) using ANOVA and Tukey’s HSD.

### 16S rRNA gene amplicon sequencing and data processing

DNA was extracted from 250 mg of soil from each sample with Power Soil DNA Isolation Kit (MO BIO Laboratories, USA). The V4 region of the 16S gene was amplified with PCR using the primers of Kozich et al. [27]. The reactions contained 22.5 μl AccuPrime *Pfx* SuperMix (Invitrogen, USA), 7.5 ng DNA, and 7.5 pmol forward and reverse primers (Integrated DNA Technologies, USA) in 25 μl final volume. Amplification was carried out in a Bio-Rad My Cycler version 1.065 thermocycler (Bio-Rad, USA) with 4 min initial denaturation at 95°C followed by 30 cycles of 20 s at 95°C, 15 s at 55°C, and 2 min at 68°C, and a final extension for 10 min at 68°C. The size and quality of the PCR products were checked using agarose gel electrophoresis. The PCR products were cleaned, quantified, pooled, and sequenced on an Illumina MiSeq instrument with a 500 cycle v2 kit (Illumina, USA) at the University of Kentucky Advanced Genetics Technologies Center according to the protocol of Kozich et al. [27]. Samples from the 7,4′-dihydroxyflavone and the naringenin experiments were sequenced separately. The sequence data is accessible at the National Center for Biotechnology Information Sequence Read Archive (www.ncbi.nlm.nih.gov/sra) under the accession number PRJNA295777.

Forward and reverse sequence reads were joined, and then sequences that were low quality, chimeric, mitochondrial, chloroplast, archaeal, eukaryotic, and unclassifiable were removed in mothur v1.34 [28] as described in the MiSeq SOP (http://www.mothur.org/wiki/MiSeq_SOP, accessed December 2014) using the SILVA alignment [29] release 119, and the Ribosomal Database Project [30] release 10. Sequences were binned to operational taxonomic units (OTUs) using minimum entropy decomposition (MED) [31] with the following settings: m = 0.0965, c = 4, M = (number of sequences in the dataset/10 000), and V = 3. A representative sequence from each OTU was classified according to the Ribosomal Database Project release 10 using mothur with 70% bootstrap cutoff.

Instead of rarefying [32], centered log-ratio (CLR) transformation [33,34] was applied to the data matrices using the compositions package [35] in R version 3.2.1 (www.R-project.org). The data matrices from the 7,4′-dihydroxyflavone and naringenin experiments contained 13 and 43 zeroes, respectively. These were replaced with ones to allow this transformation. Ordination plots were made with non-metric multidimensional scaling (NMS) in PC-ORD 6.0 (MJM Software Design, USA) with Euclidean distances: 250 runs were performed with random starting configurations in one to six dimensions with a 10^−7^ instability criterion and 500 maximum iterations with 0.2 initial step length to find the best starting configurations in each dimensionality. Statistics for the final stress for each dimensionality were obtained from 250 runs with randomized data. Dimensions were only accepted if they decreased the stress to a lower value than that from 95% of the randomized runs. Based on the results, two-dimensional solutions were selected. The final run was conducted using the determined best starting configuration. Multi-response permutation procedure (MRPP) [36] was used on the CLR transformed datasets with Euclidean distances to test the significance of the differences between the high, medium, low, and control treatments. To find differentially abundant OTUs in the treatments, we used DESeq2 (without CLR transformation) [37] as recommended by McMurdie et al. [32] with the DESeq2 package version 1.8.1 [37] in R. To account for the high number of simultaneous tests, *q*-values were calculated according to the positive false discovery rate method [38] with the smoother option in QVALUE (http://genomine.org/qvalue). The *q*-value of a test estimates the proportion of false positive findings in the dataset if tests with equal or lower *p*-values are accepted as significant.

## Results

### ATP assays

There was no significant difference in the soil ATP content between the treatments in either the 7,4′-dihydroxyflavone or the naringenin experiment (Table 1). The standard curves are shown in S1 Fig.

**Table 1:** Soil ATP concentrations, and estimated ATP extraction efficiency.

	7,4′-dihydroxyflavone (μg/g soil dry weight)	Naringenin (μg/g soil dry weight)
High	1.557±0.116	1.212±0.195
Medium	1.698±0.306	1.211±0.164
Low	1.780±0.101	0.983±0.062
Control	1.621±0.187	1.121±0.164
Extraction efficiency (%)	86.3±1.47	87.3±6.85

Numbers represent average ± SD. ATP concentrations are not corrected for extraction efficiency.

### 16S sequencing results from the 7,4′-dihydroxyflavone experiment

**Fig 1:**
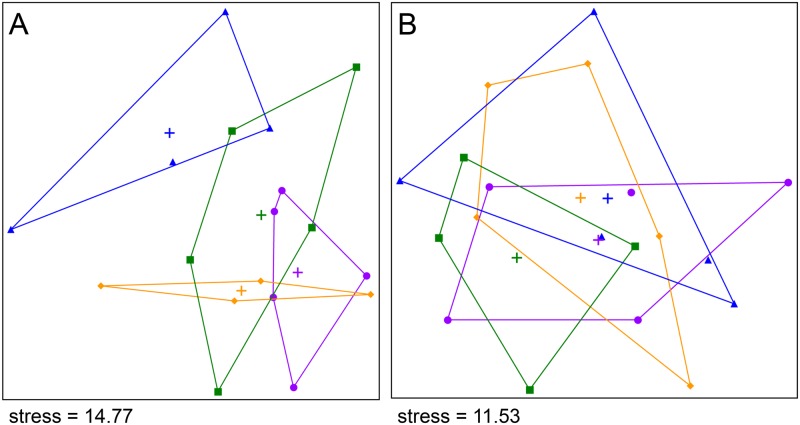
Non-metric multidimensional scaling (NMS) ordination plots. (A) 7,4′-Dihydroxyflavone experiment, (B) naringenin experiment. Points represent samples, crosses are group centroids. Samples of the same treatment are enclosed in convex hulls. Stress is calculated on a scale of 0 to 100. (Treatments: ● high, ♦ medium, ■ low, and ▲ control).

The dataset contained 117,276–359,427 sequences per sample with 253 bp average read length, which were binned to 1580 OTUs. On the NMS plot (Fig 1A) the high, medium, and low treatment samples overlapped each other, but were separate from the control samples, indicating a difference in bacterial community structure between the control and 7,4′-dihydroxyflavone treatments. This difference however, was not significant in the MRPP after correcting for multiple comparisons (Table 2).

**Table 2:** Multi-response permutation procedure (MRPP) results.

	Comparison	*A*	*p*	Corrected *p*
7,4′-dihydroxyflavone	control—low	-0.00039	0.4435	2.6608
	control—medium	0.02922	0.0446	0.2674
	control—high	0.02306	0.0333	0.1997
	low—medium	0.00497	0.3274	1.9642
	low—high	-0.00054	0.4464	2.6781
	medium—high	-0.01322	0.9229	5.5374
Naringenin	control—low	0.00323	0.3482	2.0890
	control—medium	-0.01677	0.7711	4.6265
	control—high	-0.02264	0.9584	5.7502
	low—medium	0.00028	0.4185	2.5113
	low—high	0.00403	0.3345	2.0068
	medium—high	-0.01294	0.7590	4.5542

The *A*-values are the chance-corrected within-group agreements and describe the effect size. Corrected *p*-values were calculated using the Bonferroni method.

We used DESeq2 to find differentially abundant OTUs in the control compared with the medium and high treatments. These comparisons gave the highest *A*-values in the MRPP (Table 2). The DESeq2 results including the normalized mean abundance, abundance fold change, *p*- and *q*-values, and the taxonomic classification for all OTUs are listed in S1 Table (comparing the control and high treatments) and S2 Table (comparing the control and medium treatments). There were 37 differentially abundant OTUs between the control and high treatments with *q*-values below 0.1. This *q*-value indicates the proportion of false positives if the abundances of all these OTUs are accepted as significantly different. The normalized mean abundance, abundance fold change, *p*-values, and the taxonomic classification of these 37 OTUs are shown in Table 3.

**Table 3:** DESeq2 results. Differentially abundant OTUs between the control and high treatments for the 7,4′-dihydroxyflavone experiment (*q*-values < 0.1).

	OTU#	Mean Normalized Abundance	Fold Change	*p*	Phylum	Classis	Ordo	Familia	Genus
More abundant in the control treatment	4243	142.3	0.57	0.000020	*Actinobacteria*	*Actinobacteria*	*Actinomycetales*	*Nocardioidaceae*	*Nocardioides*
	12806	197.7	0.61	0.000115	*Actinobacteria*				
	11413	338.4	0.61	0.000122	*Actinobacteria*	*Actinobacteria*	*Actinomycetales*	*Thermomonosporaceae*	
	9993	389.0	0.62	0.000314	*Actinobacteria*	*Actinobacteria*	*Actinomycetales*	*Pseudonocardiaceae*	*Pseudonocardia*
	8535	47.8	0.55	0.000381	*Actinobacteria*	*Actinobacteria*	*Actinomycetales*	*Intrasporangiaceae*	*Janibacter*
	3160	156.3	0.62	0.000501	*Actinobacteria*				
	9555	179.8	0.64	0.000525	*Actinobacteria*	*Actinobacteria*	*Actinomycetales*	*Thermomonosporaceae*	*Actinoallomurus*
	11842	691.8	0.66	0.000528	*Actinobacteria*	*Actinobacteria*	*Gaiellales*	*Gaiellaceae*	*Gaiella*
	13657	111.7	0.62	0.000748	*Actinobacteria*	*Actinobacteria*	*Gaiellales*	*Gaiellaceae*	*Gaiella*
	9030	151.0	0.65	0.001233	*Actinobacteria*	*Actinobacteria*	*Solirubrobacterales*	*Conexibacteraceae*	*Conexibacter*
	4121	319.9	0.60	0.001282	*Firmicutes*	*Bacilli*	*Bacillales*		
	9952	41.2	0.56	0.001411	*Actinobacteria*	*Actinobacteria*	*Actinomycetales*	*Nocardioidaceae*	
	5795	30.5	0.55	0.001414	*Actinobacteria*	*Actinobacteria*	*Gaiellales*	*Gaiellaceae*	*Gaiella*
	3273	163.2	0.63	0.001651	*Actinobacteria*	*Actinobacteria*	*Solirubrobacterales*		
	5985	153.5	0.64	0.001946	*Actinobacteria*	*Actinobacteria*	*Gaiellales*	*Gaiellaceae*	*Gaiella*
	10112	144.3	0.66	0.002055	*Actinobacteria*	*Actinobacteria*	*Actinomycetales*	*Nocardioidaceae*	*Kribbella*
	9557	49.6	0.58	0.002073	*Actinobacteria*	*Actinobacteria*	*Actinomycetales*	*Thermomonosporaceae*	
	7451	53.7	0.61	0.002525	*Proteobacteria*	*Alphaproteobacteria*	*Rhizobiales*		
	14169	97.4	0.63	0.002645	*Actinobacteria*	*Actinobacteria*	*Gaiellales*	*Gaiellaceae*	*Gaiella*
	5477	394.3	0.69	0.002663	*Acidobacteria*	Subdivision 17			
	3819	66.2	0.59	0.002721	*Proteobacteria*	*Alphaproteobacteria*	*Rhodospirillales*		
	14170	76.2	0.63	0.002810	*Actinobacteria*	*Actinobacteria*	*Gaiellales*	*Gaiellaceae*	*Gaiella*
More abundant in the high treatment	13198	57.6	1.91	0.000096	*Acidobacteria*	Subdivision 4			
	2945	19.0	2.05	0.000221	unclassified				
	11314	152.8	1.62	0.000637	*Acidobacteria*	Subdivision 4			
	13547	227.0	1.45	0.001289	*Acidobacteria*	Subdivision 4			
	886	28.5	1.81	0.001337	*Bacteroidetes*				
	12340	54.7	1.86	0.001555	*Bacteroidetes*	*Cytophagia*	*Cytophagales*	*Cytophagaceae*	*Adhaeribacter*
	10591	682.3	1.48	0.001584	*Acidobacteria*	Subdivision 4			
	13358	53.4	1.71	0.001622	*Acidobacteria*	Subdivision 6			
	11364	142.7	1.53	0.001717	*Acidobacteria*	Subdivision 4			
	50	30.5	1.79	0.001824	unclassified				
	13562	1270.7	1.44	0.002062	*Acidobacteria*	Subdivision 4			
	11109	57.4	1.67	0.002455	*Bacteroidetes*	*Sphingobacteriia*	*Sphingobacteriales*	*Chitinophagaceae*	*Flavisolibacter*
	12323	171.3	1.45	0.002693	*Bacteroidetes*	*Sphingobacteriia*	*Sphingobacteriales*	*Chitinophagaceae*	
	5649	203.9	1.47	0.003031	*Acidobacteria*	Subdivision 4			

Taxonomic classification of the OTUs was based on representative sequences from each OTU classified according to the Ribosomal Database Project release 10. Only taxonomic assignments with higher than 70% bootstrap support are listed. Fold change is the fold change of the normalized abundance in the high treatment compared with the control.

From the 37 OTUs, 14 had a higher relative abundance in the high treatment than in the control. Half of these OTUs were classified as *Acidobacteria* subdivision 4 (Table 3). These seven OTUs cover 18.9–22.8% of the *Acidobacteria* subdivision 4 sequences in the high treatment and 14.1–17.2% in the controls. Most of the OTUs in Table 3 that had a lower relative abundance in the high treatment compared with the control were classified as *Actinobacteria*. Six of these *Actinobacteria* OTUs belong to genus *Gaiella*, together covering 13.0–14.5% and 15.0–18.7% of the sequences from this genus in the high and control treatments, respectively. Three OTUs were classified as *Nocardioidaceae*, encompassing 37.8–47.9% and 46.3–59.5% of the sequences from OTUs classified into this family in the high and control treatments, respectively. *Thermomonosporaceae* was also represented by three OTUs, which contain 69.3–77.6% and 72.4–75.9% of the sequences from this family in the high and control treatments, respectively.

In a comparison of the control with the medium treatment, only six differentially abundant OTUs had *q*-values below 0.1 (S2 Table). Three of these OTUs (OTU# 886, 10591, and 12806) were also differentially abundant between the control and the high treatment (Table 3). Two of the other three OTUs were classified as *Micromonosporaceae* and *Acidobacteria* subdivision 16, and one could not be classified at phylum level with higher than 70% bootstrap support. Several OTUs listed in Table 3 that had significantly different abundances between the control and high treatment showed a similar response in the medium treatment compared with the control. From the *Acidobacteria* subdivision 4 OTUs listed in Table 3 that were significantly more abundant in the high treatment than in the control, OTU# 10591, 13198, and 11314 were also more abundant in the medium treatment compared with the control and had low *q*-values (0.049, 0.115, and 0.115, respectively) (S2 Table). OTU# 11842 and 14169 were classified as *Gaiella*, OTU# 4243 and 9952 were classified as *Nocardioidaceae*, and OTUs 11413 and 9555 were classified as *Thermomonosporaceae* (Table 3). Similar to the control versus high treatment comparison, these OTUs had higher relative abundance in the control than in the medium treatment and had *q*-values of 0.176, 0.209, 0.115, 0.163, 0.171, and 0.202, respectively (S2 Table).

### 16S sequencing results from the naringenin experiment

The dataset contained 149,471–339,727 sequences per sample with 253 bp average read length. The MED resulted in 1589 OTUs. Interestingly, the control samples showed no separation from the high or medium treatments in the NMS plot, but the low treatment appeared to separate (Fig 1B). This difference however, was not significant in the MRPP (Table 2). The comparisons between the low and high treatments and between the control and low treatments resulted in the largest effect sizes in the MRPP (Table 2); thus they were investigated with DESeq2 to find differentially abundant OTUs. The normalized mean abundance, abundance fold change, *p*- and *q*-values, and the taxonomic classification for all OTUs are listed in S3 Table (comparing the control and low treatments) and S4 Table (comparing the low and high treatments). Only three OTUs had significantly different abundances between the control and the low treatment and had *q*-values below 0.1 (S3 Table). One OTU was classified as *Bacillus*, one as *Gaiella*, and one was not possible to classify at the phylum level with higher than 70% bootstrap support. Comparison of the low and the high treatments resulted in four OTUs with significantly different abundances and with *q*-values below 0.1 (S4 Table). These were classified into *Oxalobacteraceae*, *Intrasporangiaceae*, *Acidobacteria* subdivision 6, and *Chitinophagaceae*.

## Discussion

Soil ATP content is a measure of the total living microbial biomass including active and dormant organisms [39]. The 7,4′-dihydroxyflavone and naringenin treatments had no significant effect on the ATP content of the soil. This suggests that the change in relative abundance of OTUs found in DESeq2 is due to a growth response of those particular bacterial groups and is not due to a general antimicrobial or growth promoting effect on the other members of the bacterial community. However we note that soil ATP content reflects the biomass of all soil organisms, including fungi, other eukaryotic microbes, and Archaea, which are not represented in our 16S sequencing results.

Naringenin has been shown to inhibit the growth of a variety of Gram positive and negative bacteria as well as *Saccharomyces cerevisiae* with minimum inhibitory concentrations ranging from 250 to 1000 μg/ml [40–42]. A broad-spectrum inhibition of growth would likely cause a decrease in soil ATP content, but this did not occur. The high flavonoid treatment solution in our experiment had a concentration of 5.45 μg/ml, which is about two magnitudes lower than the reported minimum inhibitory concentrations for naringenin. It seems likely that some root exudate flavonoids that have been considered antimicrobial based on laboratory culture studies do not function as inhibitors of bacterial growth in the soil. This is simply because the determined minimum inhibitory concentrations are higher than those expected in the rhizosphere, or they only have an antimicrobial effect adjacent to the site of exudation, such as in the rhizoplane where their local concentrations may be high.

For soils with a similar texture and pH to the Maury silt loam used in the present study, Bai et al. [25] reported a soil ATP content of 0.76–2.20 μg/g. Our results using the same extraction method also occurred within this range (Table 1). However, Bai et al. did not incubate their soils with carbon sources before extraction, whereas our samples were treated daily for 10 days with a mixture of carbohydrates and organic acids, which should promote the growth of heterotrophic microorganisms, and thus increase the microbial biomass and ATP content. In their review, Blagodatskaya and Kuzyakov [39] concluded that an ATP content below 1–2 μg/g soil is common when the soil microbial community is not activated, and that the addition of readily available substrates, such as glucose, causes several-fold increases, which implies that our results are lower than expected. A likely explanation for the relatively low ATP content of our samples is that the treatment solution added only 0.0040 mg carbon per g soil daily as glucose and other substrates, which is about three magnitudes less than the amount used in other studies to activate the soil [43,44]. The concentrations of carbon sources and the C:N ratios in our treatment solutions were closer to those used by Drake et al. [18] in their C + N treatment (carbon at 500 mg/l versus 172.3 mg/l in the present study, C:N of 10 versus 13.1 in the present study), which did increase soil microbial biomass significantly, but not several-fold as reported by Blagodatskaya and Kuzyakov [39].

7,4′-Dihydroxyflavone is known for its ability to induce the expression of *nod* genes in species like *Rhizobium leguminosarum* bv. *trifolii* [45], *Ensifer meliloti* [46], and *Bradyrhizobium japonicum* [47]. There are numerous OTUs in our dataset classified as *Rhizobium* and *Bradyrhizobium* (S1 Table), and the soil originated from a site where *M*. *sativa* had been grown for years. Therefore, it is likely that rhizobial species able to react to 7,4′-dihydroxyflavone as a *nod* gene inducer were present in the soil microbial community. However, we only found a single OTU classified as *Rhizobiales* that significantly changed (*q*-value < 0.1) its relative abundance in the high 7,4′-dihydroxyflavone treatment compared with the control (Table 3), and the abundance of this OTU decreased in the flavonoid treatment. This implies that *nod* gene induction in bacteria by 7,4′-dihydroxyflavone is not necessarily coupled with significant growth promotion. Our results concur with Hartwig et al. [5], who found that 10 μM 7,4′-dihydroxyflavone did not affect the doubling time in the cultures of *R*. *leguminosarum* bv. *trifolii* and *E*. *meliloti*.

Our results show that a large portion of the *Acidobacteria* subdivision 4 community in the soil increased its relative abundance in the high 7,4′-dihydroxyflavone flavonoid treatment. Based on culture-independent studies, *Acidobacteria* is one of the most abundant bacterial phyla in the rhizosphere [48], and subdivision 4 is among the most dominant of the 26 subdivisions in a wide diversity of soils, especially in soils with low acidity [49]. However, there are only three validly described species in this taxon [50–52], which impedes our understanding of their metabolism and activity. In clone libraries constructed from the rhizosphere of *Noccaea caerulescens* and from unplanted bulk soil that was contaminated with heavy metals, fewer clones from subdivision 4 were found in the rhizosphere compared with the bulk soil [53]. The abundance of various acidobacterial subdivisions in unplanted soil and in the rhizospheres of potato (*Solanum tuberosum* ‘Agria’), leek (*Allium porrum* ‘Kenton’), and mixed grass species dominated by *Lolium perenne* were studied in a field experiment using real-time quantitative PCR by da Rocha et al. [54]. During their four-month study, the abundances of subdivision 4 declined, but relative to the bulk soil, their abundances increased in the rhizospheres of leek and grass. The flavonoid composition of the root exudates of these plants have not been reported yet, and studies investigating the flavonoid composition of *N*. *caerulescens* shoots, potato tubers, leek bulbs, and *L*. *perenne* shoots did not find 7,4′-dihydroxyflavone [55–60]. These culture-independent studies on the abundance of *Acidobacteria* subdivision 4 point out that its growth can be influenced by plants, implying plant—microbe interactions. Our results indicate that root exudate flavonoids like 7,4′-dihydroxyflavone may be involved in mediating these interactions.

It is noteworthy that our experimental setup was not capable of distinguishing direct and indirect effects of the flavonoid treatment. Root exudate flavonoids can be modified and degraded by soil microorganisms or abiotic processes [61–63], thus some of the OTUs with significantly different abundances may have reacted to a degradation product instead of 7,4′-dihydroxyflavone itself, or to factors produced by other organisms in response to the flavonoid.

In the high 7,4′-dihydroxyflavone treatment, six OTUs classified as *Gaiella* had significantly (*q*-value < 0.1) lower relative abundances compared with the control (Table 3). This genus and the order *Gaiellales* currently have only a single described species, which was isolated from water from a deep aquifer [64]. *Gaiella* 16S rRNA sequences have been found in sediments from a thermal spring [65], in high abundance in soil [66], and in rhizosphere and root samples of rice [67]. Their presence in the rhizosphere soil and root tissues shows that some members of this genus interact with plants. According to our results, 7,4′-dihydroxyflavone from root exudates may have a role in these interactions.

In the 7,4′-dihydroxyflavone experiment, several OTUs that decreased their relative abundance in the flavonoid treatment were classified as *Nocardioidaceae* and *Thermomonosporaceae* (Table 3). These OTUs comprise a large portion of the sequences classified into these families in the dataset. Many members of these families have been isolated from soil where they can play a significant role in degradation processes and nutrient cycling [68,69]. They were found in the rhizospheres of *L*. *perenne* [70], *Solanum melongena* [71], cucumber [72], and various medicinal plants [73]. Endophytic members of *Nocardioidaceae* were isolated from the roots of wheat [74] and rice [75], whereas *Thermomonosporaceae* strains were isolated from *Aquilaria crassna* [76] and mandarin [77]. Furthermore, some of these isolates were shown to produce siderophores, which may help plant nutrient uptake, and to produce indole-3-acetic acid [73, 76, 77], and therefore potentially promote plant growth. Isolates related to *Thermomonosporaceae* were also obtained from the outer cells of the nitrogen-fixing nodules of *Casuarina equisetifolia* and were demonstrated to be able to fix atmospheric nitrogen [78], whereas other *Thermomonosporaceae* strains were shown to increase nodule biomass, nitrogen fixation rate, and to promote the growth of soybean when co-inoculated with *Bradyrhizobium japonicum* [79]. *Nocardioides* endophytes from wheat roots were also proven to be beneficial to plants as they express antifungal activity against different plant pathogens and reduce disease symptoms [80]. Therefore, it appears that some soil-dwelling members of *Nocardioidaceae* and *Thermomonosporaceae* are part of the rhizosphere microbial community of certain plants and that some colonize root tissues as neutral or plant growth promoting endophytes. However, it is currently unknown what chemical signals are involved in host recognition or induction of the colonization of plant tissues. Our results suggest that root exudate flavonoids like the 7,4′-dihydroxyflavone are potential signaling candidates influencing these plant—microbe interactions.

In the naringenin experiment, we found no significant difference in the soil bacterial community structure between the control and flavonoid treatments. Nor was there a gradually increasing response with the increasing flavonoid concentration in the treatment solution. Instead, we found the largest difference between the low and the high treatments, but there were very few differentially abundant OTUs. Longer application times or higher treatment rates may be required to study the effect of naringenin on soil bacterial community structure in a rhizosphere model system.

## Conclusions

We investigated the effect of 7,4′-dihydroxyflavone and naringenin on the total soil microbial biomass and the soil bacterial community structure in rhizosphere model systems that approximate the root exudation of *M*. *sativa* seedlings. Neither 7,4′-dihydroxyflavone, nor naringenin had an effect on the soil microbial biomass. Despite its antibacterial properties in laboratory culture studies, naringenin did not appear to inhibit microbial growth in the soil. In the rhizosphere model systems, members of *Acidobacteria* subdivision 4, *Gaiella*, *Nocardioidaceae*, and *Thermomonosporaceae* changed their relative abundance due to 7,4′-dihydroxyflavone exudation. Members of these taxa are known to interact with plants, but the signaling compounds regulating these interactions are currently unknown. Our results indicate that 7,4′-dihydroxyflavone could be among these compounds, and future studies should investigate the effect of 7,4′-dihydroxyflavone and other root exudate flavonoids on the activity of these bacteria in the rhizosphere and root tissues.

## Supporting Information

S1 Fig Standard curves of the ATP assays. (TIFF)

S1 Table DESeq2 results comparing the control and high treatments from the 7,4′-dihydroxyflavone experiment. (XLSX)

S2 Table DESeq2 results comparing the control and medium treatments from the 7,4′-dihydroxyflavone experiment. (XLSX)

S3 Table DESeq2 results comparing the control and low treatments from the naringenin experiment. (XLSX)

S4 Table DESeq2 results comparing the low and high treatments from the naringenin experiment. (XLSX)
