# Supplementary figures and images for: The Effect of Root Exudate 7,4′-Dihydroxyflavone and Naringenin on Soil Bacterial Community Structure
Source: PLoS One. 2016 Jan 11;11(1):e0146555. doi: 10.1371/journal.pone.0146555 (PMC4709137; doi:10.1371/journal.pone.0146555)

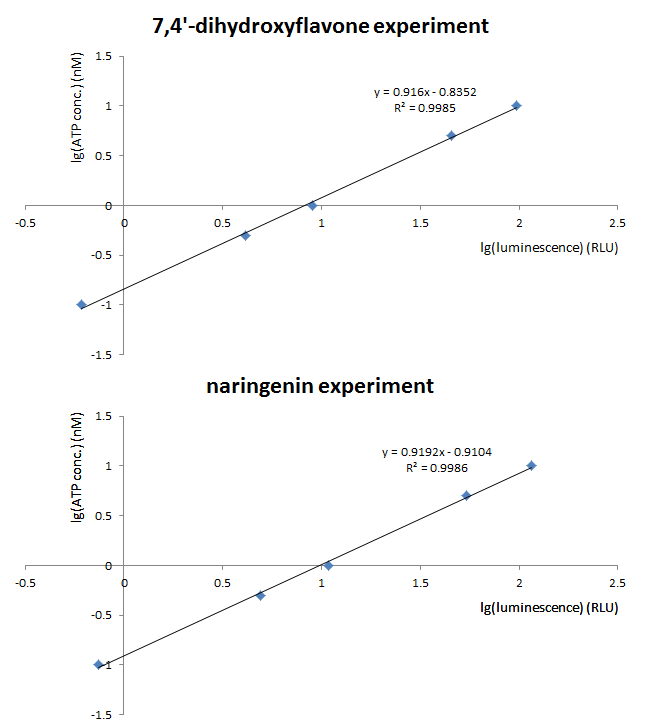

Supplement: S1 Fig — (TIFF) [file pone.0146555.s001.tiff]
